# Health conditions in land transport workers and climate change. Exploratory systematic review

**DOI:** 10.47626/1679-4435-2024-1268

**Published:** 2024-09-24

**Authors:** Wilder Alfonso Hernández-Duarte, Deisy Paola Guerrero-Calderon, William Jesús Duarte-Gómez, Angie Natalia Fonseca-Paipa, Monica Rodríguez-Muñoz, María Alejandra Tellez-Morales, Laura Camila Cubillos Garnica

**Affiliations:** Corporación Universitaria Minuto de Dios (UNIMINUTO), Bogotá, Colombia

**Keywords:** climate change, health conditions, systematic reviews as topic, transportation, cambio climático, condiciones de salud, revisión sistemática como asunto, transporte terrestre

## Abstract

A systematic exploratory review was carried out to describe the influence of climate change on the health conditions of land transport drivers, both physical and mental. Additionally, actions for the prevention of these complications are described. For the review, several databases, such as Science Direct, MEDLINE, Springer, PubMed, Redalyc, EBSCO, SciELO, and Scopus, were examined. There was the need to extend the search timeframe from 5 years to 10 years. The studies found consisted mainly of review articles, showing an emphasis on public health and a high frequency of possible physical effects on the cardiovascular and respiratory systems. At the mental level, the presence of cases of anxiety, depression and stress were mentioned. As for alternatives for the prevention of these effects, the importance of creating public policies for health promotion and disease prevention was highlighted. It is important to increase scientific production in the field of occupational safety and health and applied studies.

## Abstract (pt)

Se realizó una revisión exploratoria sistemática con el fin de describir la influencia del cambio climático en las condiciones de salud de los conductores del transporte terrestre, a nivel físico y mental. Adicionalmente, se describen acciones para la prevención de estas complicaciones. Para la revisión, se examinaron diversas bases de datos, como Science Direct, MEDLINE, Springer, PubMed, Redalyc, EBSCO, SciELO y Scopus. Se identificó la necesidad de aumentar el rango de tiempo de la búsqueda, que pasó de 5 años a 10 años. Los estudios encontrados fueron principalmente de revisión y contaban con un énfasis en salud pública; además, se destacó una alta frecuencia de posibles efectos a nivel físico en los sistemas cardiovascular y respiratorio. A nivel mental, se planteó la presencia de casos de ansiedad, depresión y estrés. En cuanto a alternativas para la prevención de estos efectos, se resaltó la importancia de la creación de políticas públicas de promoción de la salud y prevención de la enfermedad. Es importante incrementar la producción científica en el campo de la seguridad y salud en el trabajo y de estudios aplicados.

## INTRODUCTION

Over the last decade, it has been observed that climate has undergone significant variation, as indicated in the study by Pallmall^1^ a situation that has been mainly generated by human activity and consumption of fossil fuels, particularly oil and coal, which emit carbon dioxide (CO_2_), the gas that produces the greenhouse effect, a phenomenon that affects the entire population worldwide.

Due to the industrial revolution in the 1980s and current technological advances, there has been a global increase in gas emission, derived from different human activities such as tree feeling and crops of rice and other foods. Furthermore, Pallmall^1^ highlights that deforestation and maintaining animals that contribute to gas emission from their excreta are the main causes of increased greenhouse effect and climate change; moreover, the transport sector was found to be one of those that has contributed to aggravate this issue, as stated by Pico.^2^

In the labor context, climate change has currently increased heat stress, as observed by the International Labor Organization in a 2019 report showing that this condition will affect productivity in agriculture and construction. It is also important to bear in mind that workers from the land transport, sports, tourism sectors are also exposed, reducing their capacity of adapting physiologically to high temperatures and temperature changes in rainy periods, among others.^3^ In countries such as Colombia, with regard to the land transport sector, these variations lead to landslides and damages on roads, both main and side ones. Therefore, areas with a higher population and a heavier vehicle traffic are affected by the deficits in the transport system and in its operations, increasing costs of downtime and making land transport drivers divert to longer and more tiresome routes that favor exposure to factors such as prolonged postures, as shown by the Colombian Ministry of Transportation^4^ in its 2017 report on climate change and risk management.

Despite not describing a relationship with climate change, Ahumada & Ramirez^5^ conducted an analysis of the working conditions of transport workers in the city of Mexico between 1985 and 1988, which found the presence of respiratory, hearing, sensory, musculoskeletal, and gastrointestinal disorders in this sector.

Moreover, the Pan American Health Organization^6^ reported that the land transport sector, such as other economic sectors, has been impacted by effects of climate change, including storms, heat waves, and sea level rise, factors that lead travel routes and paths to deteriorate overtime, increasing drivers’ exposure to factors that affect their physical and mental health conditions. “Although heatwaves associated with climate change are not as destructive as other hazards, they can cause morbidity and mortality that are not always immediately noticeable because of several causes, including the lack of surveillance systems for occupational diseases.”^6^ Additionally, as stated by Colombia’s Ministry of Transport,^4^ it is worth considering that this sector fosters economy, since it ensures the supply of food, medications, and essential goods.

The present analysis aims to describe the circumstances that affect the health conditions of land transport drivers based on their exposure to conditions modified by climate change, in addition to describing strategies or alternatives to prevent occupational diseases so as to mitigate the impacts of climate change on the health conditions of land transport drivers and take measures to improve their safety and wellbeing at work.

The general society may also benefit from this project, since it identifies the strategies that people may apply in their routine activities, in order to contribute to improvement of transport services and reduce the costs associated with occupational accidents and diseases, in addition to improvement quality of life so as to prevent the effects of climate change. Finally, this study allows for a holistic view of new field to be investigated, in which que several future projects may be developed.

## METHODS

This is a documental study based on the collection, review, analysis, and synthesis of existing documents, aiming to understand the relationship between climate change and health conditions that affect transport workers.

### DESCRIPTION OF SEARCH STRATEGY

Search keywords were defined based on MeSH terms, i.e., words standardized by the scientific community, in order to restrict information search, using the Descriptores en Ciencias de la Salud (DeCS) thesaurus (bvsalud.org).

The following words were used: cambio climático, climate change, gases efecto invernadero, greenhouse effect gases, transporte, transporte terrestre, ground transportation, seguridad y salud laboral, salud ocupacional, occupational health, seguridad, condiciones de salud, and health status.

Furthermore, the following search equations were created:

- Cambio climático and transporte terrestre and condiciones de salud and salud ocupacional;
- Condiciones de salud and transporte terrestre and cambio climático;
- Efecto invernadero and salud ocupacional and transporte terrestre;
- Climate change and land transportation and health conditions and occupational health;
- Health conditions and ground transportation and climate change;
- Greenhouse effect and occupational health and ground transportation.

After defining keywords and phrases, a search was conducted for applied studies and reviews written in Spanish and English on the following databases: Science Direct, SCOPUS, PubMed, MEDLINE, BIREME, Springer, Redalyc, and EBSCO, in order to assess what has been written about this topic in the last 5 years.

### INSTRUMENTS

Considering the strategy described in the previous section, a database was created for the present literature review, containing information of interest from each scientific document, as well data sufficient for their online consultation. The matrix had the following headings: author – title - year of publication – abstract – conclusions – database – source (name of the journal, volume, issue, pages, and DOI, as appropriate) – type of study (review and/or original) – physical health conditions – mental health conditions – recommendations for future actions to intervene in the identified conditions.

### PROCEDURE

The present study conducted an examination on the causes that impact physical and mental health conditions of land transport drivers due to climate change, in the last 5 years, followed by a profound analysis on the relationship between the aforementioned effects. Lastly, the importance and efficacy of current policies and measures designed to mitigate the impact of this issue, thus proposing future actions that improve drivers’ situation.

### ANALYSIS OF INFORMATION

The collected information was retrieved from a detailed and systematic reading of different scientific sources found in the databases from the last 5 years, collecting ideas and reasons that explain and determine the impacts of climate change in occupational safety and health of land transport drivers, all this with the purpose of achieving the general aim.

Subsequently, frequency distribution was calculated for some variables, and content analysis was performed for variables related to effects on physical and mental health and to define intervention measures, all of this with the support of Microsoft Excel.

### ETHICAL CONSIDERATIONS

In order to protect and ensure authors’ copyright, the respective citation of studies that met inclusion criteria was provided (Appendix 1).

## RESULTS AND DISCUSSION

### GENERAL CHARACTERIZATION OF THE ARTICLES

This documentary literature review conducted the collection, review, analysis, synthesis of documents found on the scientific consultation databases such as Science Direct, MEDLINE, Springer, PubMed, Redalyc, EBSCO, SciELO, and Scopus. These articles evaluated the relationship between climate change and health conditions affecting transport workers and the evolution of this relationship over time.

Considering the time search criteria and during the collection of articles, there as the need to increase the search time range from 5 years to 10 years of publication for the articles consulted in the literature review.

The present review obtained a sample of 41 scientific articles, including both review and original ones, which initially met the criteria from the search equations. These articles were retrieved from an overall sample of 48,237 documents identified in the eight scientific databases consulted for the literature review.

One the 41 article that met the general criteria were identified, they underwent a detailed review to validate compliance with specific criteria such as analysis of interaction between climate change and physical and mental state of land transport drivers; after this stage, 23 review and original articles were selected.

The classification, review, analysis, and synthesis of the articles showed that 18 of them did not strictly meet the specific search and planning criteria of the present literature review, because of the following reasons: their design were focused on public health, they were published more than 10 years ago, they did not report investigations, or they were related to economic sector akin to transport, but not exactly to land transport.

Therefore, and according to this classification and review, the final search resulted in a sample of 23 articles included in the database of the present review. Analysis and synthesis of search results revealed that land transport workers presented physical symptoms such as cardiovascular, respiratory and musculoskeletal diseases, heat strokes, malnutrition, and fatigue at some point in their employment lifecycle. Conversely, and not less important, mental symptoms were observed, such as stress, eco-anxiety, depression and anxiety, suicide, and sleep disorders.^7^

The possible causes of this finding could be secondary to emission of greenhouse gases and PM 2.5, which affect and inflame the respiratory system. Additionally, with regard to cardiovascular diseases, not using active transport such as bicycle, increased stress and anxiety, lack of workers’ adaptation, and malnutrition lead to increased risk for such diseases.

**Figure 1:**
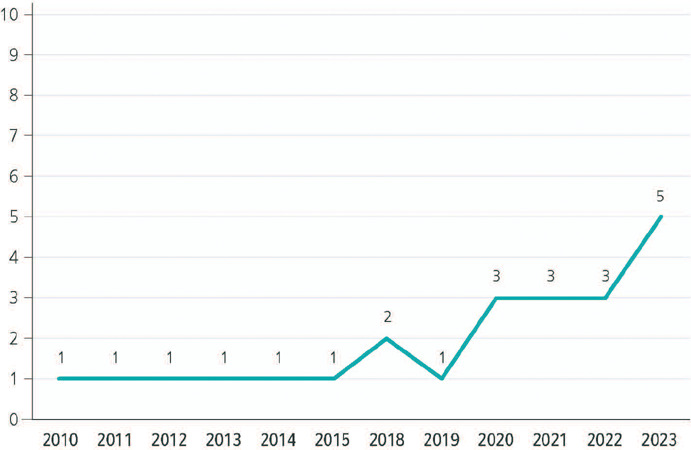
Publications identified related to the impact of climate change on the health of transport workers, by year.

Figure 1 describes the distribution of studies by year, revealing an increase in the publications after 2020, coinciding with the health emergency for COVID-19. It is also possible that the growing urgency and global attention towards climate change have fostered further research in this specific area, based on a greater global awareness on the effects of climate change, emerging policies, or significant events that have highlighted the importance of addressing transport workers’ health in this context.^8^ Among other factors, increased public concern and awareness on climate change and its health impacts may have motivated more investigators to explore this field; furthermore, as more data are collected about the actual effects of climate change on transport workers’ health, this may have generated an increased academic interest on the matter.^9^ Additionally, the occurrence of natural disasters or specific events related to climate change may have raised an renewed and urgent interest in better understanding the impacts of this phenomenon on transport workers’ health.^9^

**Figure 2:**
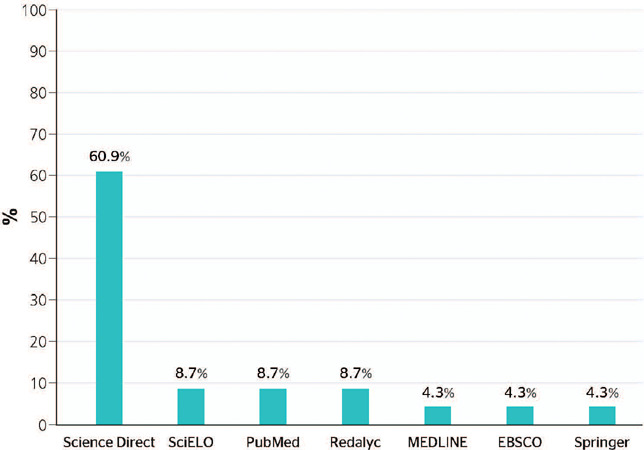
Percent distribution of documents according to database of origin.

Figure 2 shows the investigation that were found in the different scientific databases; it is possible to observe that Science Direct stands out significantly, accounting for 61% of publications. Redalyc and SciELO databases accounted for only 17% of the documents included in the present review, which reveals the scarce contribution of Latin American research to this specific field.

Considering the multidisciplinary nature of Science Direct and the specialization of the other databases in specific areas, the greatest number of articles found in Science Direct may be explained by the broad range of themes covered by this database. Since the work developed encompasses several topics from different disciplines, it is plausible that Science Direct, given its multidisciplinary nature, included a greater number of articles related to the impacts of climate change on the health of transport workers. This could partly explain the disparity in the number of articles among the different databases, since some of them may have a more specific focus on a particular discipline.

**Figure 3:**
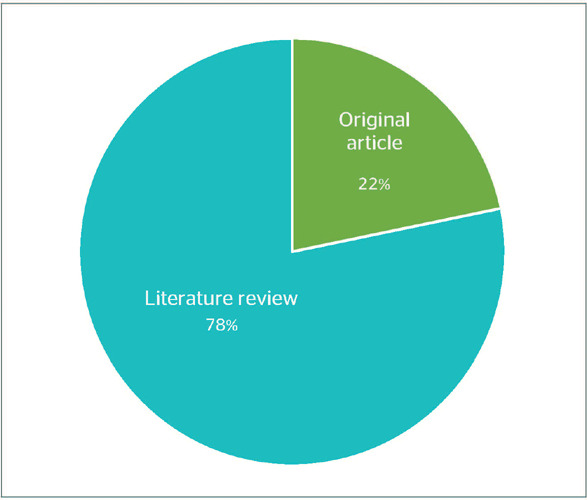
Proportion of publications identified about the impacts of climate change on the health of transport workers, according to type of study.

Figure 3 shows that the majority of publications consisted of review articles, which suggest a broad interest on the analysis and synthesis of existing knowledge on the topic. Sin embargo, the limited number of applied studies emphasized the need of further original studies, practical studies to better understand, in addition to approaching the specific effect of climate change on the health of transport workers, especially those of the land transport sector.

This information may be useful to identified gaps in the investigation and guide future efforts to address the impacts of climate change on the health of land transport workers.

### EFFECTS OF HEALTH CONDITIONS AT THE PHYSICAL LEVEL

**Figure 4:**
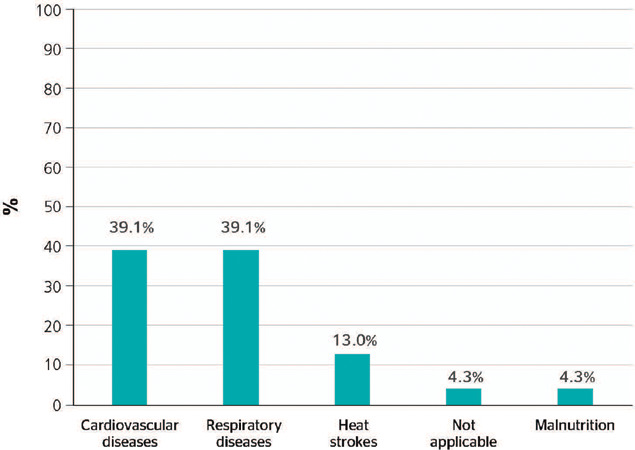
Proportion of publications about the impact of climate change on the health of transport workers, related to physical health conditions.

With regard to the effects of exposure to climate change on the physical health of transport workers, it was found that the literature reports a higher frequency of changes at the cardiovascular and respiratory level, accounting for approximately 78% of overall changes (Figure 4). Search results emphasized, for example, how respiratory infections such as tuberculosis are caused by the use of land transport in third world countries like Peru, where workers of this sector may be exposed to this disease due to the sanitary conditions to which they are possibly exposed.^10^ Another example occurred during the pandemic, as shown in a study conducted in Switzerland which also demonstrated that land transport drivers are exposed to multiple occupational risk scenarios, such as long and irregular working hours, physical workload conditions, and psychosocial factors arising from contact with passengers, in addition to being exposed to natural phenomena and physical, chemical, biological factors.^11^ Furthermore, exponential contagion of users of land transport, especially public, was evident during the severe acute respiratory syndrome (SARS-CoV2) infection pandemic; as a consequence, in this period it was necessary to create novel public policies to reduce contagion of respiratory diseases such as COVID-19.^12^

It is also worth mentioning a noticeable fact observed in the review, namely the role of air quality, since the particulate matter emitted by land transport increased morbidity and mortality in countries such as the United States.^13^ Therefore, the economic impact of implementing strategies to control emission of particulate matter and/or gases, as shown in China, where these strategies were found to be beneficial for population’s health, impacting both the health and on the economic sectors of the country.^14^

Conversely, cardiovascular diseases were also an important topic in the field of physical health conditions, with findings revealing the multiple benefits of being physically active.^15^ A study conducted in the city of Bangkok showed once more the impact of particulate matter (PM 2.5) at the economic level and at the respiratory and cardiovascular health levels, in addition to the importance of implementing novel technologies, such as electric ones.^16^ Similar findings were also observed in a systemic review conducted in Sub-Saharan Africa that compared the importance of using active transport (walking and cycling) vs. the benefits and wellbeing it brings to this population. This review emphasized that a friendly infrastructure to pedestrians, specific interventions, and culturally sensitive strategies may potentially promote active transportation and improve health in this population.^17^ Therefore, multiple articles identified recommend active transport such as bicycle, in addition to actions to reduce the use of private vehicles, thus fostering a more sustainable and healthy population.

It should also be considered that cardiovascular diseases related to climate change are mediated by air pollution,^15,18^ increased ambient temperature, vector-borne diseases, and mental health disorders.^19,20^ Hence the importance of implementing policies and conditions that allow for improving the quality of environment and population’s lifestyles.^21^ As previously mentioned, if one actually intends to reduce CO_2_ generation, it is inevitable using an active mode of transport or one that generates less impact, such as electric land transport.^22,23^

It is also worth highlighting the function and the role of healthcare workers, since, as shown by an analysis conducted in Chile in 2018, these professionals are individuals in which the society trusts; therefore, they should play an active role in the issue of climate change and health, instructing the population on health promotion and prevention, both generally and individually, about surveillance systems, disaster preparedness, or disease control.^24^ There is a clear relationship between lifestyle and climate change, which allows to address them simultaneously; therefore, it is important to adopt a multidisciplinary approach, thus having an impact on physical conditions and on daily occupations, through the promotion of sustainable occupations.^25^

The transport sector presents scenarios characterized by daily scarcity of energy and sustainable resources, a context that raises the challenge of developing public focuses and policies to reduce environmental impact,^26^ since reduction of greenhouse effect gases may prevent the advancement of climate change and secondarily bring health impacts. This fact reinforces the importance of developing sustainable environmental technologies in all possible environments (energy, construction, transport, agriculture, forestry, waste management, and environmental health),^27^ encouraging the use of more environmentally friendly in order to mitigate the impact on human health.^28^

### EFFECTS OF HEALTH CONDITIONS AT THE MENTAL HEALTH LEVEL

**Figure 5:**
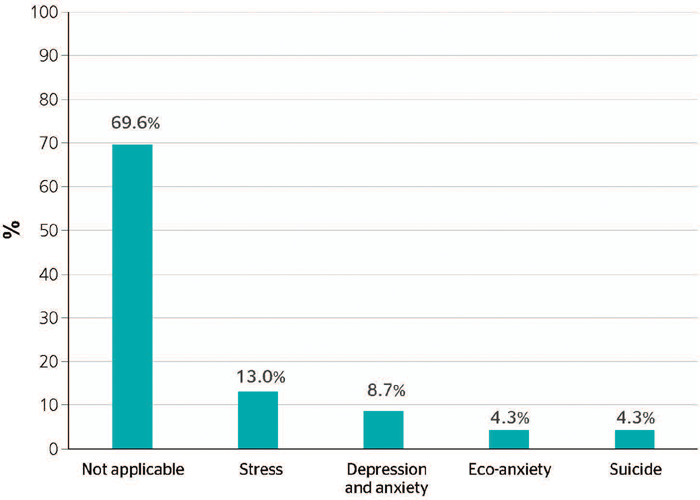
Proportion of publications about the impacts of climate change on the health of transport workers, related to mental health conditions.

In relation to mental health conditions, the search found that the most common diseases in land transport workers were depression and stress, with long working hours, exposures to dramatic climate changes and lack of infrastructure^8^ are triggering factors for workers of the sector under analysis to develop these conditions (Figure 5). These irregularities do not allow for workers to perform their functions optimally; therefore, intervening on psychosocial risk factors is a beneficial strategy.^29^

Depression usually comes with anxiety, since transport workers are a broad group that has been exposed to different occupational circumstances, an example of which was the period of COVID-19 pandemic,^11^ an event that highlighted the importance of generating future resources to improve the response to crisis such as pandemics and situations that cause adaptive changes in workers.

In turn, global warming, one of the effects of climate change affecting the earth and which has greenhouse effects as a consequence, its effects on the health of land transport workers are increasingly more adverse as this phenomenon becomes more intense,^30^ leading to high accident rates, due to heat strokes that they may suffer as an associated condition, and thus facilitate situations of stress, anxiety, and depression.^21^ Although anxiety is not a severe condition among psychological disorders, depression is, and thus suicide may be a consequence drawn from a depressive behavior in land transport workers.^30^

Mental health conditions in workers as a consequence of prolonged exposures to risk factors resulting from climate change lead to mental disorders that can even cause depressive diseases, which in turn result in possible anxiety symptoms. Conversely, it can be deduced, according to Zhao et al.,^21^ that cardiovascular diseases related to climate change are mediated by air pollution, increased ambient temperature, vector-borne diseases, and mental health disorders.

### STRATEGIES OR ALTERNATIVES FOR PREVENTION OF OCCUPATIONAL DISEASES

The results of the present review show that there is a relationship between climate change and health conditions that affect land transport workers; therefore, it is important to consider public proposals related to food hygiene in street vending places,^8^ in order to avoid direct contact with pollutant emitted by public passenger transport and by food street stalls that occasionally are alternative solutions for drivers. Conversely, it is suggested to articulate this recommendation and include it into the Occupational Health and Safety Management System (OHSMS).

Furthermore, another recommendation identified in the present literature review and analysis, according to a study based on shifting from sedentary to active transport, is implementing global public policies, articulated with non-government organizations, in order to support the shift from passive or sedentary modes of transport in large cities to modes of transport such as bicycles, thus building more cycle and pedestrian paths.^29^ It is essential to identify road infrastructure projects articulated with non-governmental organization so as to promote road improvement, thus reducing travel times and avoiding environmental secondary pollution from particulate matter. It is important to clarify that this recommendation should be articulated with the different road safety strategic plans and with the different occupational health and safety current legal regulations for the prevention of chemical, physical and psychosocial factors in each country where the same condition is present.

In face of climate change and greenhouse effect gas emission due to population growth in large cities and to use of land and passenger transport to meet the demand for transporting both merchandise and passengers, it is necessary to impose a greater control on this sector by creating new taxes designed to generate a social welfare culture.^31^

Different points of intervention may be included in the OHSMS. It is important to emphasize that actions such as implementation of preventive medicine will allow for prioritizing campaigns aimed at health promotion and disease prevention through actions that allow for workers to increase the use of active transport (such as bicycle) and reduce the use of private cars. These actions should also raise awareness on the importance of using personal protective equipment, such masks, and washing hands in case of respiratory disease outbreaks resulting from seasonal winter peaks and motivate workers to follow a healthy diet as a way to feeling well and having good health. Furthermore, it would be recommendable to emphasize the review of workers’ health state at the cardiovascular and respiratory levels through the occupational medical examinations required to organizations.

Furthermore, it bears highlighting that the identification of psychosocial risk factors plays an important role in preventing conditions that affect mental health. Therefore, there is the need to create spaces where workers can express themselves and be in work environments where dialogue and safe conditions allow workers to have a more reliable adaptation and to perform their functions optimally.

With the purpose of addressing this specific climate-change related issue in land transport workers, it is recommended to implement intersectoral work groups including the public institutional level and the private sector,^30^ leading to massive health promotion and disease prevention strategies in occupational safety and health^32^ and to reengineering actions in health system preparedness.

During the literature search, there was a predominance of information coming from studies in the field of public health, since the impact of climate change on land transport workers is an emerging theme; furthermore, as shown in the present investigation, pollution and greenhouse effect are a reality that involves all sectors, both economic and political, at an international level.

## CONCLUSIONS AND RECOMMENDATIONS

The present exploratory review highlights the higher prevalence of cardiovascular and respiratory diseases, emphasizing the exposure of transport workers to significant risks and evidencing the need of public policies aimed at reducing air pollution and promoting more active and sustainable lifestyles.

The analysis of mental health conditions in the land transport sector found literature describing disorders such as depression, stress, and anxiety among workers of this sector, factors largely influenced by prolonged working hours, exposure to climate change factors, and absence of appropriate infrastructure to cope with this change. There is an imperative need for implementing educational programs, both at business and social levels, as well as promoting strategies to prevent psychosocial factors derived from exposure to climate impact factors.

The development of educational strategies at business and social levels on the effects of climate change was proposed, in order to clearly understand how to act in case of heat strokes and suicide. These flagship programs should be mapped within the different development plans of countries worldwide that experience the present issue.

Alternatives were described to prevent occupational diseases so as to mitigate the impacts of this phenomenon in the health conditions of land transport drivers, through a documental study based on the analysis and synthesis of existing documents. There is the need to consider and update activities involving occupational medicine, disease prevention, and health promotion, as well to reinforce the importance of implementing other modes of transportation and sustainable sources, such as bicycle, walking, and electric energy, in order to prevent certain diseases presented by transport workers.

In the context of strategies and/or alternatives for disease prevention, importance is given to the creation of public policies of health promotion and disease prevention directed to the land transport sector and that enable to raise awareness and proactively intervene to mitigate the impacts derived from climate change and greenhouse gas effect emission. Additionally, it is also important to raise awareness of workers of the land transport sector about comprehensive self-care for physical and mental health, in addition to understanding how companies can support this process by strengthening occupational wellness initiatives within the framework of management systems.

As recommendation for future studies and investigations, it is suggested to compare the existing public policies in terms of the already known health impacts and their effectiveness. Furthermore, another aspect to consider is broadening the search with regard to cardiovascular diseases as a consequence of climate change in transport workers, since, despite the existence of studies on the topic, there is scarce literature explaining or discussing the physiopathology and the relationship between these two phenomena.

It is recommended to broaden research about other psychological diseases that affect land transport workers, considering the mental component and the issue of eco-anxiety, a term not usually found in the literature but that stands out in the land transport sector.

## Appendix

**Appendix 1:** Synthesis of articles that met the criteria to be assessed

Authors	Title	Year of publication	Physical health conditions	Mental health conditions	Recommendations of future actions to intervene on the conditions identified
Garaycochea & Ticona^10^	Rutas de transporte público y situación de la tuberculosis en Lima, Perú	2015	Respiratory	Not applicable	Public transport should be considered in the ongoing fight against tuberculosis (TB). It is accepted that Lima’s public transport need to be remodeled, due to different factors, to which it would be convenient to add the public health variable, related with decreased risk of exposure to TB by reducing the most used types of vehicles (“kombis”); reordering routes, since many of these take similar paths; not allowing for vehicles to carry more passenger than it would be acceptable, with priority in the routes -higher probability of TB transmission. Moreover, public transport vehicles should be used as spaces of disease prevention and health promotion.
Vajjarapu et al.^8^	Evaluating climate change adaptation policies for urban transportation in India	2020	Malnutrition	Stress	To implement resilience strategies de within the community interested in using passenger public transport services in the country. To identify road infrastructure projects articulated with non-governmental organizations so as to promote road improvement, thus reducing travel times and avoiding environmental secondary pollution from particulate matter. Furthermore, to implement a public food hygiene policy in street vending places, in order to avoid direct contact with pollutants emitted by public passenger transport in food street stalls in India.
Zapata-Diomedi et al.^15^	Shifting car travel to active modes to improve population health and achieve transport goals: A simulation study	2023	Cardiovascular diseases	Not applicable	According to a study based on shifting from sedentary to active transport, it is recommended to implement global public policies, articulated with non-governmental organizations, in order to support the shift from passive or sedentary modes of transport in large cities to modes of transport such as bicycles, thus building more cycle and pedestrian paths.
Remy & Guseva Canu^11^	The impact of the SARS-CoV-2 pandemic on health and working conditions of Swiss bus drivers	2023	Respiratory diseases	Depression and anxiety	To generate more spaces of participation to syndication of companies, so as to increase workers’ employment and health guarantees. Implementing occupational health prevention strategies in articulation with associated entities, in order to deal with the different biological risk factors that may arise over the next years .
Chavanaves et al.^16^	Health impacts and costs of fine particulate matter formation from road transport in Bangkok Metropolitan Region	2021	Cardiovascular diseases	Not applicable	Jointly and temporarily removing (scrapping) all vehicles responsible for PM2.5 emission to the environment.
Villanueva et al.^30^	Cambio climático y salud	2019	Heat strokes	Suicide	To design educational strategies at the business and social levels on the effects of climate change, in order to clearly understand how to act in case of heat strokes and suicide.
Choma et al.^13^	Health benefits of decreases in on-road transportation emissions in the United States from 2008 to 2017	2021	Respiratory diseases	Not applicable	To implement stringent policies for of GHG emissions caused by land passenger transport.
Khurshid et al.^31^	Driving towards a sustainable future: Transport sector innovation, climate change and social welfare	2023	Respiratory diseases	Not applicable	To determine penalties to companies that generate more emissions that it is legally allowed, in order to generate social welfare, and to promote innovations to reduce GHG in order to foster social welfare.
Jones^7^	The health impacts of climate change: why climate action is essential to protect health	2022	Heat strokes	Eco-anxiety	To raise awareness of land transport drivers about the identification of risk factors related to natural phenomena and about the identification of psychological signs of anxiety and posttraumatic stress.
Tian et al.^14^	Economic impacts from PM2.5 pollution-related health effects in China’s road transport sector: A provincial-level analysis	2018	Respiratory diseases	Stress	To apply modes of shared transport to reduce the use of vehicles that use fossil fuels and contribute to reduction of GHG, as well as strategies to make better use of spare time.
Beltrán et al.^29^	Psychosocial Factors and Occupational Diseases in Workers of an Urban Public Transportation System, Mexico	2011	Not applicable	Stress	To perform training sessions with workers on mental health to improve the quality of work climate.
Musau et al.^32^	Association between transport-related physical activity and wellness in sub-Saharan Africa: A systematic literature review	2023	Cardiovascular disease	Not applicable	To implement interinstitutional and private investment alliances to create an infrastructure for non-motorized and/or active transport (transport-related physical activity). To develop joint institutional boards aimed at identifying sociocultural beliefs, mobility behavior, and their implications for health outcomes, highlighting the need for context-specific interventions.
Khan & Burdzik^28^	Measurement and analysis of transport noise and vibration: A review of techniques, case studies, and future direction	2023	Respiratory diseases	Not applicable	To temporarily encourage and structure the use of environmentally-friendly vehicles, e.g., electrical ones, through the implementation of public transport policies articulated with national development plans (countries worldwide).
Jacobsen et al.^20^	Climate change and the prevention of cardiovascular disease	2022	Cardiovascular diseases	Not applicable	To design strategies to prevent cardiovascular diseases associated with air pollution and heat.
Zhao et al.^21^	Global climate change and human health: Pathways and possible solutions	2022	Cardiovascular diseases	Depression and anxiety	To promote healthy lifestyles and to prepare the health system for climate change.
Shaw et al.^22^	Health co-benefits of climate change mitigation policies in the transport sector	2014	Cardiovascular diseases	Not applicable	It is recommended to implement the sustainable development goals, in articulation with public policies to mitigate the use of different modes of land transport.
Miriam Levi, Tord Kjellstrom, and Alberto Baldasseroni	Impact of climate change on occupational health and productivity: a systematic literature review focusing on workplace heat	2018	Cardiovascular diseases	Not applicable	To design policies to reduce the impact of emotions and climate change on nephropathy and on respiratory and cardiovascular diseases. Moreover, to include renal screening tests (BUN and creatinine), spirometry, and lipid profile in periodic and entrance medical examinations.
Palmeiro-Silva et al.^24^	La amenaza del cambio climático a la salud de la población y la necesidad urgente de actuar	2020	Cardiovascular diseases	Not applicable	To promote campaigns that allow for improving the environment, generating environmental health policies.
Diaz & Richardson^25^	Occupational therapy’s contributions to combating climate change and lifestyle diseases	2021	Cardiovascular diseases	Not applicable	To create occupational health strategies to promote lifestyle changes and to evaluate the impact of physical environment on health.
González Sánchez et al.^23^	El cambio climático y sus efectos en la salud	2013	Heat strokes	Not applicable	To prepare workers with personal protective equipment that helps mitigate climate change-related diseases.
López-Olmedo et al.^12^	Revision rápida: probabilidad de contagio por infecciones respiratorias agudas en el transporte público y medidas para mitigarlo	2020	Respiratory diseases	Not applicable	Drivers should remain silent. When shouting, singing, or speaking, they may emit particles at an amount sufficient to transmit respiratory diseases, especially in confined, poorly-ventilated, and densely occupied spaces such as subway cars or trucks.
Barbero & Tornquist^26^	Transporte Y Cambio Climático: Hacia Un Desarrollo Sostenible Y De Bajo Carbono	2012	Respiratory	Not applicable	To promote energy efficiency, within a scenario of a more expensive energy supply, in order to reduce the costs and dependence on fuels, as well as its influence on the mitigation of climate change.
Tirado Blazquez^27^	Cambio climático y salud. Informe SESPAS 2010 Climate change and health. SESPAS report 2010	2010	Respiratory	Not applicable	It was suggested that intervention measures to reduce gas emissions affect organization systems such as transport, housing, or power supply are more effective if they consider aspects of adaptation and mitigation of climate change. In this context. policies and measures have been promoted to integrate adaptation and mitigation of climate change into development plans and sector decision making. The benefit of this integration includes promotion of sustainable investing and reduction of vulnerability to climate impacts, both current and future.
